# Clinical Outcomes and Shoulder Kinematics for the “Gray Zone” Extra-articular Scapula Fracture in 5 Patients

**Published:** 2020-02-07

**Authors:** Jyoti Sharma, Candice Maenza, Andrea Myers, Erik B. Lehman, Andrew R Karduna, Robert L Sainburg, April D Armstrong

**Affiliations:** 1 Department of Orthopaedics, Geisinger Health System/Holy Spirit, Camp Hill, PA, USA; 2 Department of Neurology, Penn State Health Hershey and Penn State College of Health and Human Development, Hershey, PA, USA; 3 Department of Orthopaedics and Rehabilitation, Penn State Health Hershey and Penn State College of Medicine, Hershey, PA, USA; 4 Department of Public Health Sciences, Penn State College of Medicine, Hershey, PA, USA; 5 Department of Human Physiology, University of Oregon, Eugene, OR, USA

**Keywords:** Scapula fracture, Scapulohumeral rhythm, Glenohumeral rhythm, Motion analysis, Shoulder kinematics, Extra-articular scapula fracture

## Abstract

**Aims:**

There is a subset of scapula fractures, which can be considered in the “gray zone,” where treatment guidelines are not clear-cut, based on published literature. Our paper presents the outcomes of five such scapula fractures treated non-operatively.

**Methods:**

Adult patients who had been treated non-operatively at our institution for an isolated scapula fracture from 2003–2012 were found using Current Procedural Terminology (CPT) codes. Based on injury imaging, these five patients had scapula fractures in the “gray zone.”

Subjects completed questionnaires [Simple Shoulder Test (SST), PROMIS Global Health Scale vs 1.1, PROMIS SF vs 1.0 Physical Function 12a, and the American Shoulder and Elbow Surgeons Score (ASES)] and physical exams were performed to assess range of motion and strength. Glenohumeral kinematics were obtained via motion analysis using the Trackstar 6 Degree of Freedom (DOF) motion tracking system by Northern Digital Incorporated.

**Results:**

All subjects were right hand dominant. 3/5 fractures involved left, non-dominant, scapulae. Motion analysis demonstrated similar recruitment of the scapula during the glenohumeral rhythm for the fractured shoulders compared with the same arm of age matched control subjects. No significant differences occurred in either range of motion (ROM) or scapula-humeral coordination when comparing uninjured scapulae to the same arm of age matched control subjects.

**Conclusions:**

All subjects’ demonstrated acceptable clinical outcomes when treated non-operatively. Minor differences were seen in subjective surveys. However, the kinematic analysis showed no differences in measured scapula-humeral rhythm or range of motion. It is proposed that immediate controlled range of motion and rehabilitation be considered in these patients and could be the focus of a larger prospective study.

**Level of Evidence:**

Level IV (Case Series).

## Introduction

Scapular fractures are typically caused by high-energy impacts and accompanied by more severe vital organ injuries. They are relatively uncommon comprising only 1% of all fractures and 5% of all shoulder fractures [1].

The treatment of the majority of extra-articular scapular fractures has been traditionally non-operative because the rich soft tissue envelope surrounding the scapula provides mechanical stability, nutrients needed for fracture healing, and a cushion that mitigates a certain extent of deformities [1]. However, it has been traditionally suggested that surgical treatment be considered for scapular neck and body fractures that have substantial angulation (> 45 degrees) on the scapular Y view, a glenopolar angle of less than 23 degrees in the coronal plane, or superior suspensory shoulder complex injury. Translation of the lateral border of greater than 2 centimeters on the AP view (medialization) or 1.5 centimeter with angular deformity of greater than 30 degrees on the sagittal Y view has also been described as an indication for surgery [2]. Although these treatment guidelines are available, a number of studies have reported satisfactory clinical results following non-operative treatment of the scapular fractures that fall into the surgical indication criteria mentioned previously [3–12]. Surgical fixation of scapular fractures often involves a large surgical exposure with potential complications such as infection, nerve injuries, and fixation failure. What is not available in the published literature is a rigorous kinematic analysis of scapula-humeral coordination in extra-articular scapula neck and body fractures treated non-operatively, where the indication for operative versus non-operative treatment is not as clear; the “gray zone.” These include fractures that have a component of displacement and that meet some, but not all of the conventional surgical criteria. It is possible that with a closer look at the three dimensional kinematics of the scapula-humeral coordination one could find abnormalities that otherwise would be underappreciated based on physical examination alone. The purpose of this study was to investigate the clinical outcomes of non-operative treatment in patients who sustained a scapular neck fracture with body involvement that fall into this “gray zone” and to analyze the glenohumeral kinematics of the affected scapula in comparison with the same arm of age-matched control participants and with the unaffected scapula. Based on clinical experience, we hypothesized that 1. these patients would perform well based on traditional clinical assessment but that 2. detailed, three-dimensional motion analysis might reveal substantial range of motion limitations, and scapulohumeral discoordination.

## Materials and Methods

### Patient identification

After receiving approval by our institutional review board, a database, using Current Procedural Terminology (CPT) codes was created which included all adult patients (> 18 years of age) who had been treated by the Penn State Health Milton S. Hershey Medical Center Bone and Joint Institute for a scapula, clavicle, and/or glenoid fracture from January 1, 2003 to December 31, 2012. A total of 1,539 patients were identified. Patients were then excluded if they had any concomitant injury in the same upper extremity, a scapula fracture that was treated surgically or other known shoulder pathology. Patients with isolated scapula fractures treated non-operatively were identified (n=321). Available imaging was reviewed in the hospital imaging system GE PACS (General Electric’s picture archiving and communication system). Patients with original injury radiographs of the shoulder or scapula and a CT scan of either the chest (including the scapula of interest) or upper extremity were further selected (n=180). Images were reviewed and those patients with isolated glenoid, acromion, or coracoid fractures were excluded (n=30). Patients with bilateral scapula fractures were excluded (n= 5). Non-displaced scapular fractures were also excluded (n=38) as were intra-articular glenoid fractures (n=16). A total of 92 patients were identified that met the inclusion criteria. Forty one patients could not be contacted due to incorrect phone numbers or disconnected telephone lines. Of the remaining 51 patients, only 5 met our criteria as falling into the “gray zone” based on injury imaging and were willing to participate. Five age and sex matched controls were contacted for the control group and found through a database of healthy volunteers maintained at our institution. As stated above, our rigorous selection criteria in this low-incidence disorder resulted in a small, but somewhat consistent group of patients, in terms of degree of damage.

### Clinical assessment

Subjects were consented for the motion analysis study by a research assistant. Each subject completed questionnaires about their perceived shoulder function and received a clinical examination of their shoulder to assess their range of motion and strength by the research assistant. The questionnaires included the Simple Shoulder Test (SST), PROMIS Global Health Scale vs 1.1, PROMIS SF vs 1.0 Physical Function 12a, and the American Shoulder and Elbow Surgeons Score (ASES).

### Imaging

**Figure 1:**
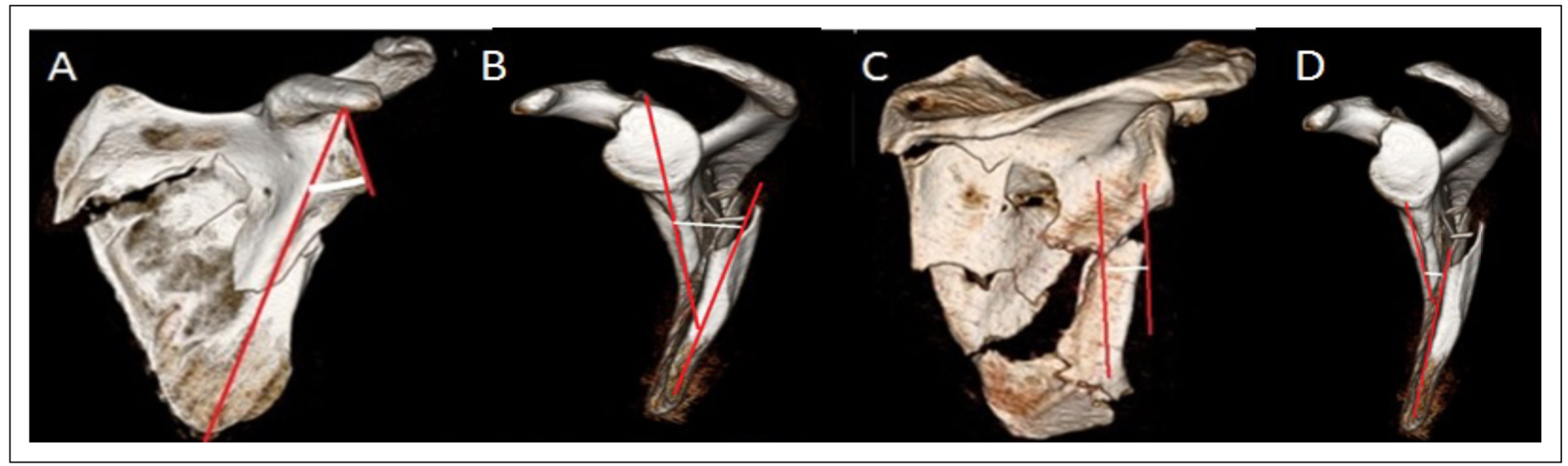
Representative measurement methodology. Measuring glenopolar angle (GPA) (A) by measuring the angle between the line connecting the upper and lower poles of the glenoid and the line connecting the upper pole of the glenoid with the inferior scapular angle. Angulation displacement (AD) measurement (B) for scapula fractures where a line is drawn through the proximal fragment in parallel with the cortices just proximal to the fracture and a second line is drawn through the distal fragment in parallel with the cortices just distal to the fracture on the scapular Y view. The subsequent angle is measured. Medial lateral displacement measurement (MLD) (C) measured on an AP image by drawing two vertical lines, one from the lateral most side of the superior fragment and the other from the lateral most side of the inferior fragment. Intervening distance is measured. Anterior posterior displacement (APD) measured on a scapular Y view (D) by measuring the distance between the anterior cortices of both the proximal and distal fragments.

**Figure 2:**
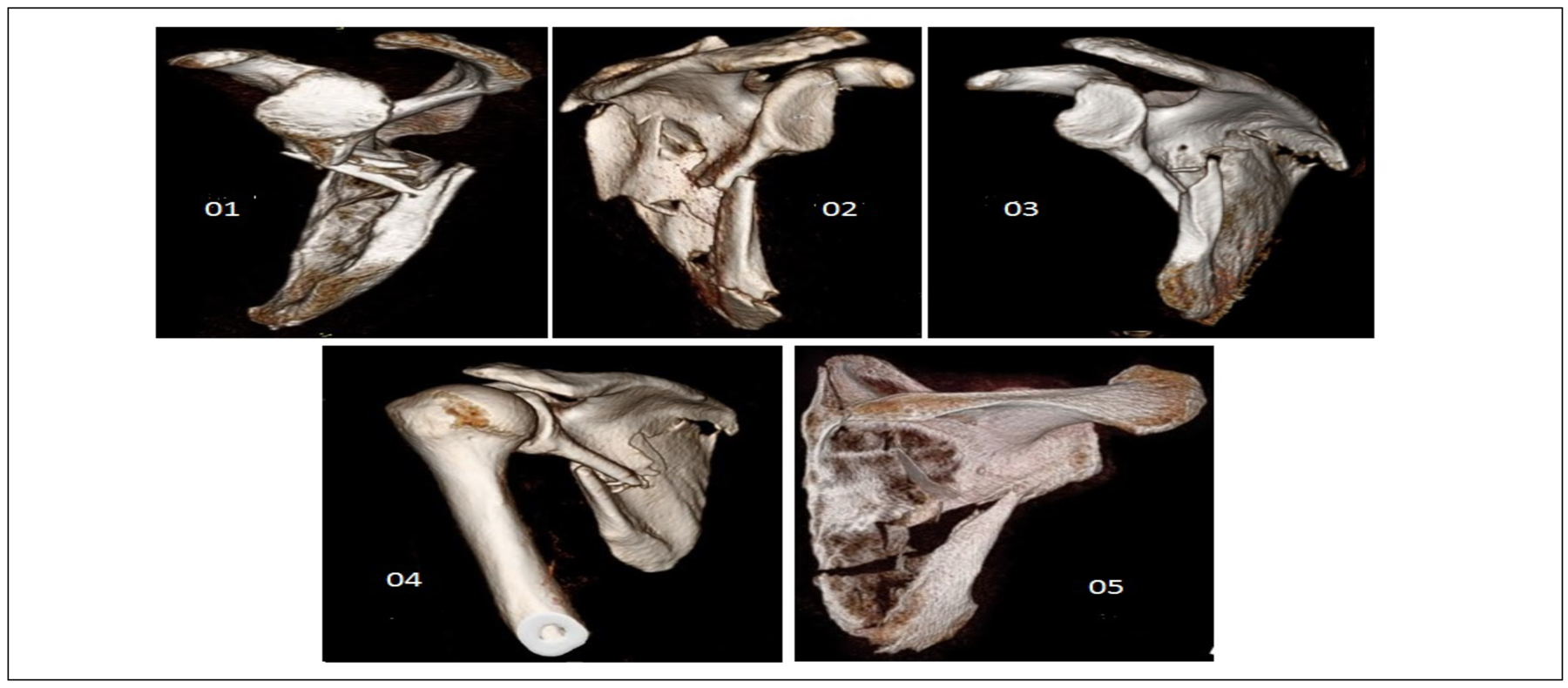
Select injury 3D reconstruction images of the scapula fractures of the five subjects in this study who were all treated non-operatively at our institution.

Patients had to have original injury films of the affected shoulder, scapula, and a CT scan of either the chest (including the scapula of interest) or upper extremity. Three dimensional reconstructions of the scapula, with humerus subtraction, were then obtained in order to standardize orientation of the scapula and create meaningful comparisons between patients [13]. We measured medial lateral displacement (MLD) and glenopolar angle (GPA) in the coronal plane and angular deformity (AD) and anterior posterior displacement (APD) in the sagittal plane, as shown in Figure 1 [2]. We defined potentially clinically important MLD as 20 millimeters or more, GPA as less than 23 degrees, APD of greater than 15 millimeters with AD greater than 30 degrees, or AD greater than 45 degrees based on indications for surgery previously described in the literature [14,15]. Figure 2 illustrates 3D reconstruction images of the scapula fractures of the subjects in this study.

## Motion Analysis

### Apparatus

**Figure 3:**
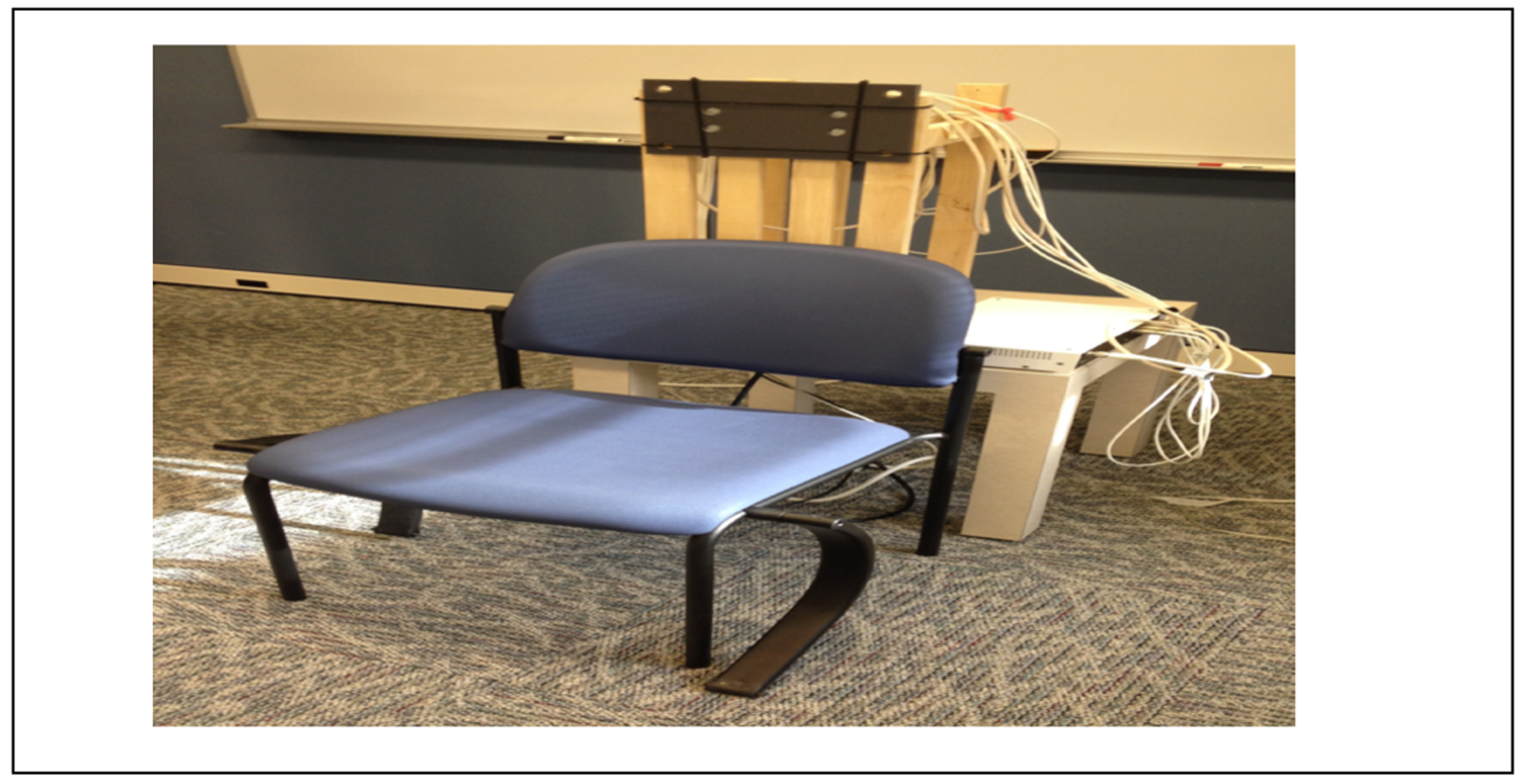
Motion analysis apparatus consisting of the transmitter mounted to a rigid base and a chair for the subjects.the five subjects in this study who were all treated non-operatively at our institution.

**Figure 4:**
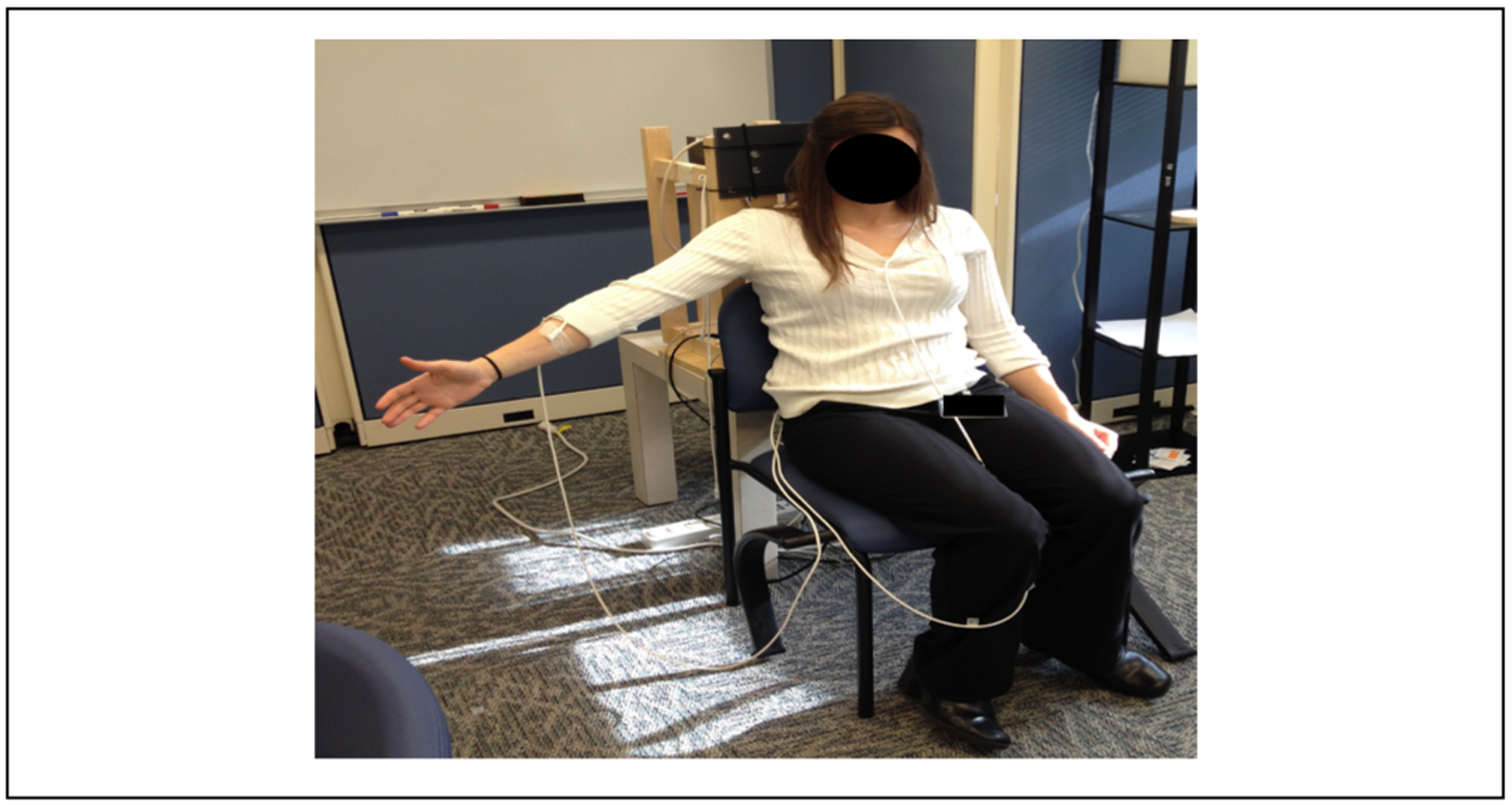
Subject positioning for motion analysis. Four sensors were attached to the subject and secured with a pre-wrap tape.

All kinematic recordings were conducted according to the recommendations of International Society of Biomechanics (ISB) [6]. Kinematic data of arm and scapula movements were collected using 4 6-DOF magnetic sensors (Ascension TrackStar). The sensors provided position (3 DOF) and orientation (3 DOF) with respect to the magnetic transmitter. A global coordinate system was established by mounting the transmitter on a rigid wooden base, such that the z-y plane aligned with the sagittal plane and the x-y plane aligned with the coronal plane of the subject. Subjects sat in a chair facing away from the transmitter (Figure 3). Scapula movements were measured using the acromial method [9] in which a sensor was directly attached to the broad, flat surface of the posterior-lateral acromion with double sided tape. This area was identified by the investigator following the spine of scapula to the flat area acromion proximal to the origin of the deltoid. This method is shown to be within 5 degrees of agreement of a more invasive bone screw method for humerus angle elevations below 120 degrees [16,17]. A second magnetic sensor was placed on the thorax at the level of T3 with double-sided tape. Arm movements were measured by a magnetic sensor placed on the lateral mid-shaft of the upper arm and another sensor placed on the forearm [18]. Thus, 3-D, high-resolution motion of the scapula, humerus, and forearm were recorded. We slightly modified the recommendations by Wu, et al. [19] and digitized the following points on each body segment: C7 and C8 vertebral spinal process, Sternal notch (SN), Xyphoid process (XP), The Inferior angle of the scapula, The acromial angle of the scapula, The root of the spine of the scapula, and the coracoid process, The head of the humerus, The lateral and medial epicondyles (most caudal points on each), The ulnar styloid process (most caudal point). All sensors were secured using a pre-wrap tape (Figure 4). The kinematic data was sampled at 116 Hz to obtain position and orientations of individual sensors. Custom computer algorithms for experiment control and data analysis were written in REAL BASIC (REAL Software, Inc.) and MATLAB (Mathworks Inc.).

### Digitization and anatomical motion

The kinematic data from the sensors were converted to anatomical motions using axes derived from digitized bony landmarks as shown in Table 1. From these bony landmarks, the axis attached to the individual segments was computed (Table 2).

### Joint angle calculations

All joint angles were computed according to the ISB recommendations [19].

### Quantifying scapular engagement

During arm elevation, the humerus and scapular move together, with a greater contribution from the scapula with higher elevation angles of the humerus. Previous research has noted that during the first 30 degrees of humeral elevation, a “scapular setting phase” occurs, in which most motion is due to glenohumeral rotation, with little contributions from the scapula [19]. Thus, two phases of scapulohumeral coordination can be identified. Scapular-humeral joint coordination is different in these two phases of motion, and the progressive transition between these two phases can indicate shoulder health. Further, the scapular-humeral joint coordination among individuals with shoulder pathologies is expected to be different in each of these phases. To quantify the transition between two phases of motion, a piecewise linear function with one transition point was fitted to the shoulder elevation angle with dependent variable being the humerus elevation angle. This led to an optimization problem with 5 parameters: two parameters each for slope and intercept and one called transition point that quantified transition from one phase of movement to another. For fitting, the transition point was varied between 0 and 1 in increments of 0.01, and two lines were fit to data on the left and right of the transition point. The set of parameters that gave the least mean-squared error was chosen as the best fit solution. This yielded two linear regressions, characterizing each phase of motion. The slope of these regressions reflected the scapulohumeral coordination in each phase. We thus compared these slopes for patients with scapular pathology and age, sex, and arm matched control subjects. This approach of quantifying scapulohumeral coordination through regression analysis into two phases of motion provides a unique and rigorous analysis of scapulohumeral coordination.

### Statistical analysis

All data were summarized prior to analysis with means, medians, and standard deviations or frequencies and percentages. The distribution of continuous variables was assessed using histograms, normal probability plots, and box plots. The clinical data were skewed and not normally distributed and the sample size was small, therefore, nonparametric tests were employed. This included a Wilcoxon Rank Sum test to compare the control to the scapula fracture group and the group with a right-side fracture to the group with a left side fracture in the scapula fracture group. A Wilcoxon Signed Rank test was also used to compare the active to passive measures within the scapula fracture group.

All kinematic data were quantified by comparing the damaged arm of patients to the same arm of control participants, who were matched for age, sex and handedness. Comparison between the arms of patients was not done because of reported differences between shoulder postures and motion in the dominant and non-dominant arms [20]. In addition, changes in range of motion of the arm with scapular damage can affect trunk motion and thus motion of the opposite arm. Kinematic analysis quantified scapulohumeral rhythm as the slope between the scapula elevation angle and the humeral elevation angle, relative to the scapula at two phases in movement, identified by the optimization algorithm described above (see quantifying scapular engagement). Mixed factor ANOVA used group (patient, control) as the between subjects factor and movement phase as the within subjects factor. Statistical significance was set at p=0.05, and all analyses were performed using SAS version 9.4 (SAS Institute, Cary, NC).

## Results

Table 3 illustrates the subject demographics and the raw data collected from the questionnaires. Note that each study subject is color and number matched to his age and sex matched control subject. All subjects were male. The average age of the scapula fracture patient at the time of injury was 60.4 years and at the time of motion analysis was 65.4 years. The average age of the control group was 62.4 years (p =1.0). Mean follow up time from the year of injury was 5.6 years (range 3.2–9.2 years).

Three patients had left scapula fractures while two had right sided injuries. For 2 patients, the fracture was on the dominant side (02 and 05). One patient (02) reported pain in the shoulder at time of motion analysis. This same patient also reported the lowest scores in 4/5 of the questionnaires. The median SST score was 11.0 for the patients versus 12.0 for the controls (p =0.366), with 12 being a perfect score. The average ASES score for the patients was 84.7. For controls, the average ASES score was 94.5 when calculated for the bilateral upper extremities. A perfect score is 100 and indicates better function and patient satisfaction. The other three surveys performed were the global health physical function, health mental, and physical function. Raw values from the questionnaires were converted in T scores based the scoring PROMIS Global Short Form. A high score represents more of the concept being measured. Therefore, a person with T-scores of 60 is one standard deviation better (more healthy) than the general population [21]. The median T-score global health physical function score for the patients was 50.0 versus 57.7 for the controls (p=0.832). Similarly, for the health mental score, the median T-score for the patients was 50.8 while it was 67.6 for controls (p=0.018). Median physical function T-score for the patients and controls was 52.4 and 52.4, respectively (p=0.525).

Tables 4A and B show the physical exam findings in regards to shoulder range of motion and strength and Table 5 summarizes these findings. Examination of overall shoulder strength did not demonstrate any deficits except for in scapula fracture patients 01 and 02. Patient 01 demonstrated diffuse weakness on exam in the setting of no pain and decreased overall motion. Patient 02 showed some weakness on exam with external rotation, however, demonstrated full strength with the remainder of testing. This patient did report 3/10 pain.

Table 6 illustrates the radiographic measurements related to the scapula fractures in the subjects in our study. Medial lateral displacement (MLD), angular deformity (AD), anterior posterior displacement (APD), and glenopolar angle (GPA) were measured for all fractures in the five subjects using the techniques shown in Figure 1. No fracture met standard operative criteria when all four measurements were taken into consideration, therefore falling into the “gray zone.”

### Kinematic analysis

**Figure 5:**
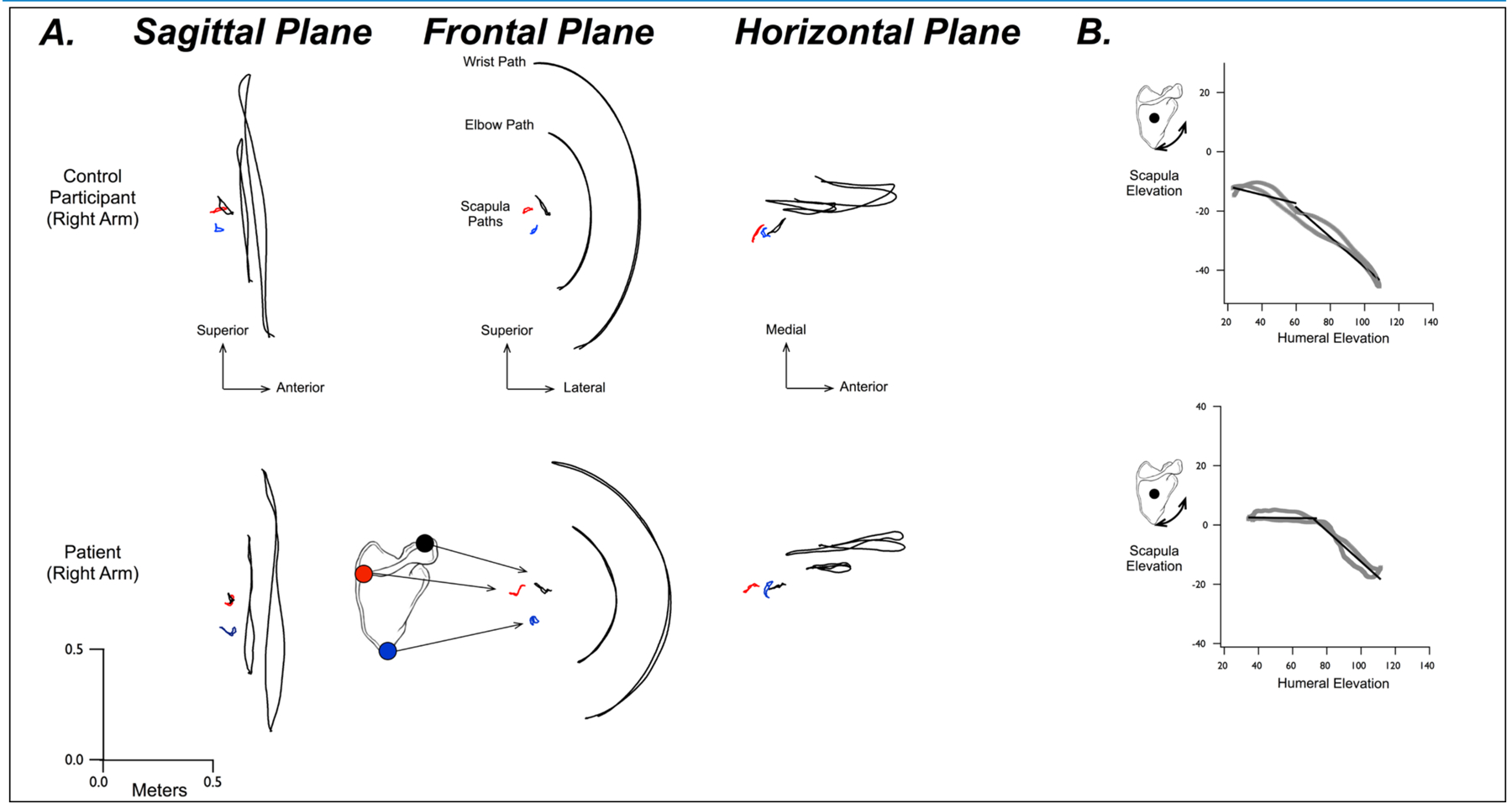
A. Paths of the wrist, elbow, and 3 locations on the scapula, the inferior angle (blue), root of the scapular spine (red), and acromioclavicular joint. Typical movement paths for the right arm of a control participant (top) and a patient with a fractured right-scapula (bottom). Paths are shown in the sagittal, frontal, and horizontal planes. B. Scapula elevation angle vs Humeral elevation angle for the movement shown in A. Solid lines show linear regressions derived from optimization analysis for the initial and final phases of motion.

Figure 5 shows typical right-arm movements for one patient (fractured side) and one control participant. Sagittal plane, frontal plane, and horizontal plane paths are shown for the wrist, elbow, and for 3 digitized points on the scapula. As can be seen in Figure 5A, these points form a triangle in the frontal plane, but overlap in the other planes. The lack of overlap between the scapular paths with the elbow and wrist paths in the sagittal and frontal plane indicates the oblique orientation of the motion, which was a maximum upward and downward motion of the shoulder with the outstretched arm, in the plane of the scapula, or roughly 10° to 20° anterior to the frontal plane. Note that the paths are very similar between the control and the patient.

We quantified scapulohumeral coordination as the relationship between scapular elevation and humeral elevation in each of two phases of motion, a scapular setting phase (phase 1) and the phase following this (phase 2). In order to objectively and rigorously quantify scapulohumeral coordination, these two phases were determined by using an optimization algorithm that fit a linear regression to each phase, adjusting the slopes and intercepts of each regression line, as well as the transition point between them. The algorithm found the minimum difference (error) between the fit lines and the empirical data. This analysis is depicted in the plots in Figure 5B. The continuous relationship between scapular elevation and humeral elevation is depicted in gray for the movements shown in Figure 5A. The dark lines show the linear regressions before and after the transition point defined by our optimization. As reflected in these plots, the slope of these lines is substantially different, indicating a transition from largely humeral motion (phase 1) to substantial recruitment of scapula (phase 2).

**Figure 6:**
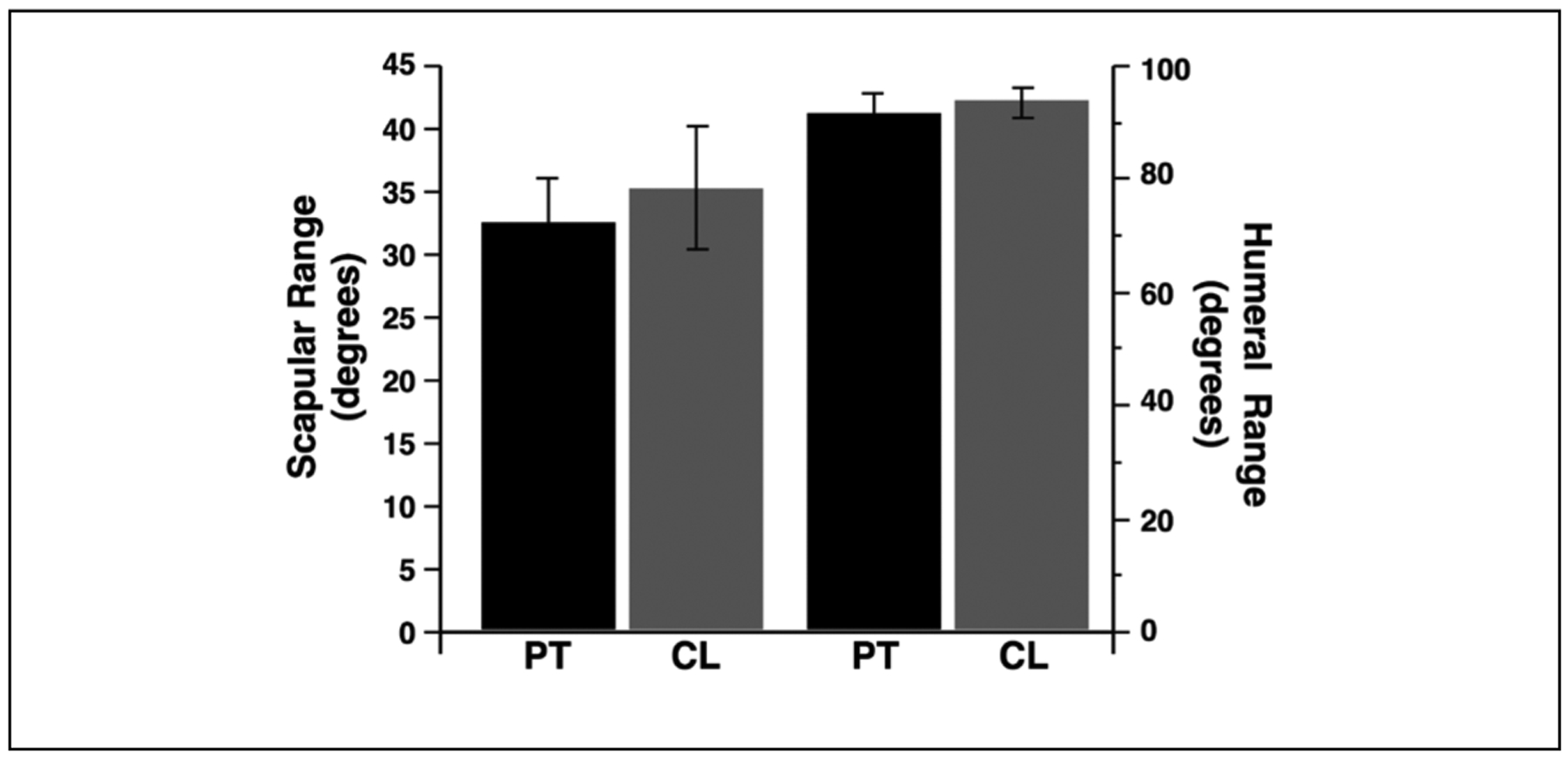
Mean (±SE) across patients (PT) and Control Subjects (CT) for initial phase of motion (Left) and final phase of motion (Right).group. No statistically significant differences were found, likely due to small sample size. Overall, scapula fracture patients maintained similar ROM compared to uninjured side and to the control group.

Figure 6 shows the average slope of scapulohumeral coordination for each phase of motion (phase 1 and phase 2), separated by patients (PT) and control participants (CT). As reflected by the data in Figure 5, regardless of whether the shoulder was previously fractured, the slope in Phase 1 was substantially smaller than in Phase 2. In addition, fractured scapulae shoulders in patients showed a trend toward steeper coordination in phase 2, reflecting slightly greater scapular contributions to motion. However, this trend was not significant. We conducted a mixed factor ANOVA for the slope of scapulohumeral coordination, with phase (1, 2) as the within subject factor and group (control, patient) as the between subject factor. Our results showed a main effect of phase [F(1,16.16) = 18.47, p < 0.01], but not for group [F(1,12.63) = 1.70, p = 0.21], nor was there an interaction between these factors [F(1,16.16) = 0.34, p =0.57].

**Figure 7:**
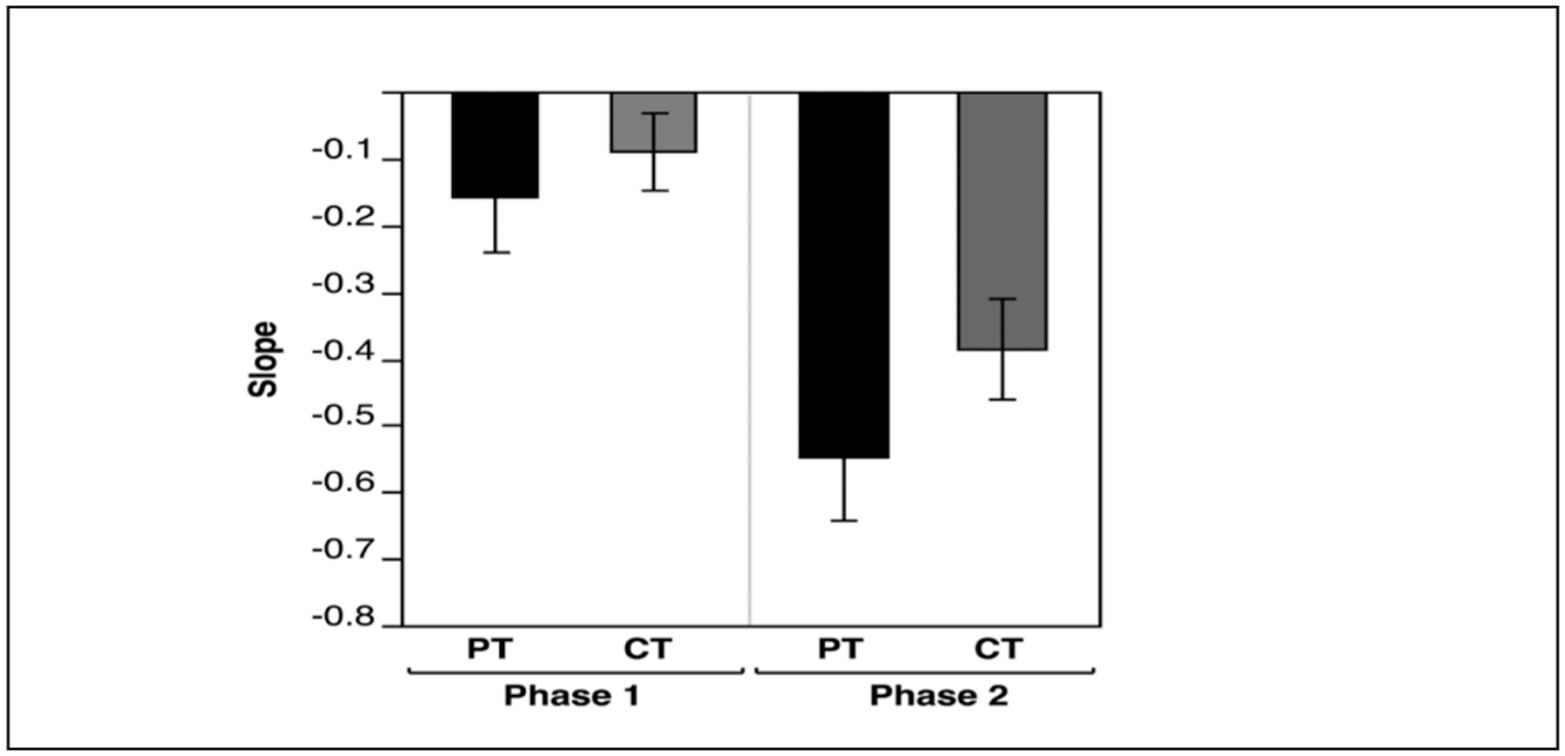
Mean (±SE) across patients (PT) and Control Subjects (CT) for scapular range of motion (left axis and left 2 bars), and humeral range of motion (right axis and right 2 bars).

Figure 7 (Left) shows scapular range of motion and Figure 7 (Right) shows humeral range of motion, in control subjects as compared with the fractured shoulder of patients. There was no significant difference in either measure between the fractured shoulder of patients and the matched shoulder of control subjects [Scapular ROM: F(1,1) = 0.18, p =0.67, Scapular ROM: F(1,1) = 0.16, p =0.69]. These results rigorously demonstrate that scapula-humeral coordination and range of motion during maximum range arm lifting movements is not affected by gray-zone scapular fractures, in the chronic phase of the disorder. This may contrast with clinical judgements regarding scapulohumeral rhythm and joint range, neither of which rigorously and quantitatively assess range of motion and coordination.

## Discussion

In our clinical experience, it was our impression that extra-articular scapula fractures that met some of the reported radiographic parameters for considering surgical intervention could still do well from a clinical standpoint despite non-operative treatment. Thus, we designed this study of high-resolution 3D motion analysis of scapulohumeral coordination and range of motion during instructed maximum range arm lifting, in order to help to assess the effects of extra-articular scapular fractures on range of motion and coordination. We developed a rigorous analysis of scapulohumeral rhythm, employing an optimization algorithm to identify the slope and intersection of two linear regressions, thus identifying two phases of scapulohumeral coordination. Our sample size was limited by the low-incidence of this disorder and by our rigorous selection criteria that limited our analysis to patients with gray-zone scapular damage. This refers cases in which the indication for operative versus non-operative treatment is not clear; including fractures that have a component of displacement and that meet some, but not all of the conventional surgical criteria. We only included patients who were treated non-operatively.

Clinically, our non-operatively treated scapula fracture patients appeared to do well overall. We found no significant differences in survey scores between the subjects and control participants. Therefore, these scapula fractures that fell into a “gray zone” showed no apparent clinical consequences from non-operative treatment when looking at their outcome scores and average strength and range of motion results. This would suggest that our guidelines for treating scapular neck fractures with body involvement either operatively or non-operatively may warrant further investigation. These patients could have been treated surgically based on previously published criteria; however, with such treatment risks of infection, nerve damage, and hardware failure, to mention a few, it would be important to reexamine our criteria to warrant such risk. None of these patients, except scapula fracture patient 02, had any pain at evaluation and all demonstrated pain free functional motion, despite a healed malunited fracture. No patient developed a nonunion of their fracture. These findings are in contrast to those published by Nordqvist that scapular neck fractures treated non-operatively were more likely to have fair or poor results [10]. The risk of undertaking surgical intervention must be reconciled with evidence that outcomes may be satisfactory with non-operative treatment.

Surprisingly, rigorous analysis of scapulohumeral coordination and range did not reveal any significant differences in these measures between our control group and our patient group (fractured scapulae). While scapulohumeral rhythm changed, as expected in the early versus late phase of motion, coordination in each phase was similar between groups. The switch point signifies the recruitment of the scapula by the humerus during the glenohumeral rhythm. Usually this occurs at around 30 degrees of humeral elevation. After that, a relatively consistent 2:1 ratio is seen on average for glenohumeral motion to scapulothoracic motion [13]. Our findings (Figure 6) show a trend toward higher contributions of scapula to the scapulothoracic motion in the second phase of motion (after the switch point), but this trend did not reach significance. These patients did not show abnormal coordination nor limitations in range of motion that would be predictive of activity limitations. These patients who received non-surgical treatment for gray-zone scapular fractures do not experience limitations in scapula-humeral coordination nor changes in active range of motion. Thus, non-surgical treatment appeared adequate to preserve normal coordination and range of motion. We feel that this study is important because it brings into question the surgical criteria that are being utilized to indicate for surgical intervention. Despite meeting some but not all of the surgical indications for surgery, these patients did well both clinically and also showed no functional compromise when looking at the detailed scapulohumeral kinematics for the patient. It is not clear in the literature the priority of the surgical criteria or how many of these criteria should be met when trying to decide whether or not a patient should have surgery.

Clear guidelines for rehabilitation after non-operative treatment for scapula fractures is lacking in the literature. In general rehabilitation after a scapula fracture tends to be rest, followed by gentle physical therapy and range of motion exercises three to six weeks after the injury to allow for bone healing. Pendulum exercises and pain control modalities are often started immediately [22,23]. There is literature to suggest that immediate physical therapy following proximal humerus fractures provides better results compared to starting therapy 3 weeks after injury [24]. It should be stressed that while our results indicate no differences in patients and controls with regard to scapulohumeral coordination, our task was a slow raising the arm through its range of motion. It is important to note that we did not test kinematics during a range of activities, and it remains possible that the effect of the injury on scapulohumeral kinematics may become more significant during tasks requiring rapid coordinated motion, and with significant resistance such as required for many work and leisure activities including lifting, throwing, and tool manipulation.

One of the main limitations of our study was our sample size in this low-incidence disorder. However, we believe that this was at least partially compensated by the consistency of the sample population that was dictated by our strict inclusion criteria, and by the rigor of our kinematic analysis. Given that the vast majority of the patients tend to be trauma patients at our level one trauma center, they do not always follow up in our health care system. Consequently, out of the 92 patients that met our inclusion criteria. In addition, 41 could not be contacted due to change or disconnection of phone numbers and addresses. Also, no radiographs were obtained at the time of this study, so fracture healing and residual deformity could not be assessed. Strengths of this study, however, were the use of a true control group to compare the injured shoulder of patients to the same arm of control subjects, and rigorous kinematic quantification of scapulohumeral coordination and range of motion.

## Conclusions

Overall, we support our hypothesis that patients with displaced fractures of the scapula neck that fall into the “gray zone” for treating operatively versus non-operatively demonstrate acceptable clinical outcomes, in this small sample group, in the long term when treated non-operatively. Minor differences were seen in overall motion and subjective surveys. However, we did not support our second hypothesis that three-dimensional motion analysis would reveal substantial range of motion limitations, and scapulohumeral discoordination since no significant differences were found in our kinematic analysis of range of motion and scapulohumeral coordination when comparing to the control group. It is proposed that immediate controlled range of motion and rehabilitation could be considered in these patients. The intent of this study is to highlight that future investigation into the operative criteria for scapula fractures and rehabilitation protocols may be warranted.

## Figures and Tables

**Table 1:** Axes derivation from digitized bony landmarks.

	Point 1	Point 2	Point 3	Point 4
**Thorax**	C7 vertebra	T8 vertebra	Sternal notch (SN)	Xyphoid process (XP)
**Scapula**	Acromial Angle (AA)	Root of scapular spine (SP)	Inferior angle (IA)	Crociod Process
**Humerus**	Head of Humerus (HH)	Lateral epicondyle (LE)	Medial epicondyle (ME)	
**Forearm**	Ulnar Styloid (US)	Lateral epicondyle	Medial epicondyle	

**Table 2:** Computation of the axis attached to the individual segments.

Segment	X- axis	Y- axis	Z- axis
**Thorax**	Xth: Perpendicular to C7, T8, and SN plane	Yth: Cross of Z and X	Zth: C7 to T8
**Scapula**	Xsc: SP to AA	Ysc: Perpendicular to AA, SP, and IA plane	Zsc: Cross of X and Y
**Humerus**	Xh: Cross of Y and Z	Yh: Perpendicular to ME-LE-HH plane	Zh: Midpoint of ME and LE to HH

**Table 3:** Patient demographics and questionnaire outcomes. [Simple Shoulder Test (SST), PROMIS Global Health Scale vs 1.1, PROMIS SF vs 1.0 Physical Function 12a, and the American Shoulder and Elbow Surgeons Score (ASES)]. Color coordination identifies matched subjects and controls.

STUDY NUMBER	AGE AT VISIT	GENDER	INJURED SIDE	DAYS BETWEEN INJURY AND VISIT	DOMINANT SIDE	CURRENT PAIN IN SHOULDER 0–10	SST	GLOBAL HEALTH PHYSICAL FUNCTION T-score	GLOBAL HEALTH MENTAL T-score	PHYSICAL FUNCTION T-score	ASES
01 SCAP FX	80	M	LEFT	1588	RIGHT	0	11	61.9	53.3	53.8	90
02 SCAP FX	51	M	RIGHT	1648	RIGHT	3	4	42.3	45.8	43.5	46.7
03 SCAP FX	69	M	LEFT	3346	RIGHT	0	12	61.9	53.3	52.4	100
04 SCAP FX	57	M	LEFT	1184	RIGHT	0	9	50	44.9	45.8	86.6
05 SCAP FX	70	M	RIGHT	2426	RIGHT	0	12	47.7	50.8	53.8	100
01 CONTR	75	M	NA		RIGHT	0	12	61.9	67.6	44.3	100-B
02 CONTR	44	M	NA		RIGHT	0	12	57.7	67.6	59.5	100-B
03 CONTR	64	M	NA		RIGHT	3	12	44.9	67.6	52.4	85-R 85-L
04 CONTR	58	M	NA		RIGHT	2	11	57.7	62.5	55.8	85-R 90-L
05 CONTR	71	M	NA		RIGHT	0	11	54.1	53.3	52.4	100-B

**Table 4A:** Physical exam at time of motion analysis demonstrating range of motion. Values represent degree measurements. T = Thoracic. L = Lumbar.

STUDY NUMBER	RIGHT FORWARD ELEVATION ACTIVE	RIGHT FORWARD ELEVATION PASSIVE	LEFT FORWARD ELEVATION ACTIVE	LEFT FORWARD ELEVATION PASSIVE	RIGHT EXTERNAL ROTATION AT SIDE ACTIVE	RIGHT EXTERNAL ROTATION AT SIDE PASSIVE	LEFT EXTERNAL ROTATION AT SIDE ACTIVE	LEFT EXTERNAL ROTATION AT SIDE PASSIVE	RIGHT INTERNAL ROTATION	LEFT INTERNAL ROTATION
01 SCAP FX	130	140	130	135	35	40	35	40	BELT	TL
02 SCAP FX	145	150	175	180	75	75	80	85	TL	T5
03 SCAP FX	160	165	160	165	50	55	45	50	T7	T5
04 SCAP FX	170	175	165	170	50	55	50	55	T5	T5
05 SCAP FX	170	175	170	175	60	65	60	65	T7	T7
01 CONTR	155	165	170	175	60	65	55	60	T5	T5
02 CONTR	180	180	180	180	45	50	45	50	T5	T5
03 CONTR	170	175	170	175	85	85	80	85	T5	T5
04 CONTR	160	170	170	175	40	45	40	45	TL	T7
05 CONTR	175	175	175	175	45	50	45	50	T5	T5

**Table 4B:** Physical exam at time of motion analysis demonstrating strength testing of the shoulder based on the Muscle Grading System (ASIA).

STUDY NUMBER	RIGHT EXT ROT AT SIDE (1–5)	LEFT EXT ROT AT SIDE (1–5)	RIGHT JOBE (1–5)	LEFT JOBE (1–5)	RIGHT BELLY PRESS	LEFT BELLY PRESS	RIGHT LIFT OFF TEST	LEFT LIFT OFF TEST
01 SCAP FX	4	4	4	4	NEG	NEG	UNABLE	UNABLE
02 SCAP FX	4	4	5	5	NEG	NEG	NEG	NEG
03 SCAP FX	5	5	5	5	NEG	NEG	NEG	NEG
04 SCAP FX	5	5	5	5	NEG	NEG	NEG	NEG
05 SCAP FX	5	5	5	5	NEG	NEG	NEG	NEG
01 CONTR	3	3	3	3	NEG	NEG	NEG	NEG
02 CONTR	5	5	5	5	NEG	NEG	NEG	NEG
03 CONTR	5	5	5	5	NEG	NEG	NEG	NEG
04 CONTR	5	5	5	5	NEG	NEG	NEG	NEG
05 CONTR	5	5	5	5	NEG	NEG	NEG	NEG

**Table 5:** Summary of comparisons in motion for scapula fracture patients and control group. No statistically significant differences were found, likely due to small sample size. Overall, scapula fracture patients maintained similar ROM compared to uninjured side and to the control group.

Control vs. Scapula Fracture (N=10)						
Shoulder Motion	Control (N=5)		Scapula Fracture (N=5)		Control vs. Scapula Fracture P-value	
	Right	Left	Right	Left	Right	Left
**Average Active FE (range)**	170.0 (160.0**–**175.0)	170.0 (170.0**–**175.0)	160.0 (145.0**–**170.0)	165.0 (160.0**–**170.0)	0.243	0.130
**Average Active ER (range)**	45.0 (45.0**–**60.0)	45.0 (45.0**–**55.0)	50.0 (50.0**–**60.0)	50.0 (45.0**–**60.0)	0.916	0.916
**Average Passive FE (range)**	175.0 (170.0**–**175.0)	175.0 (175.0**–**175.0)	170.0 (165.0**–**175.0)	165.0 (150.0**–**175.0)	0.234	0.219
**Average Passive ER (range)**	50.0 (50.0**–**65.0)	50.0 (50.0**–**60.0)	55.0 (55.0**–**65.0)	55.0 (50.0**–**65.0)	0.916	0.916

Scapula Fracture, Left vs. Right Injured Side (N=5)				
Shoulder Motion	Side	Left (N=3)	Right (N=2)	P-value
**Average Active FE (range)**	Right	160.0 (130.0–170.0)	157.5 (145.0–170.0)	1.0
	Left	160.0 (130.0–165.0)	172.5 (170.0–175.0)	0.149
**Average Active ER (range)**	Right	50.0 (35.0–50.0)	67.5 (60.0–75.0)	0.139
	Left	45.0 (35.0–50.0)	70.0 (60.0–80.0)	0.149
**Average Passive FE (range)**	Right	165.0 (140.0–175.0)	162.5 (150.0–175.0)	1.0
	Left	165.0 (135.0–170.0)	177.5 (175.0–180.0)	0.149
**Average Passive ER (range)**	Right	55.0 (40.0–55.0)	70.0 (65.0–75.0)	0.139
	Left	50.0 (40.0–55.0)	75.0 (65.0–85.0)	0.149
Shoulder Motion	Side	Active (N=5)	Passive (N=5)	P-value
**Average FE**	Right	160.0 (145.0–170.0)	165.0 (150.0–175.0)	0.063
	Left	165.0 (160.0–170.0)	170.0 (165.0–175.0)	0.063
**Average ER**	Right	50.0 (50.0–60.0)	55.0 (55.0–65.0)	0.125
	Left	50.0 (45.0–60.0)	55.0 (50.0–65.0)	0.063

* Median (Q1–Q3 range), Wilcoxon Rank Sum Test

**Table 6:** Measurements of the scapula fractures in the 5 subjects. Measurements were taken using the original injury films and 3D reconstructions. Yellow boxes denote measurement values of significance that meet or are close (3 mm or 3 degrees) to surgical intervention based on previously published criteria. No fractures reached all 4 criteria for surgery when measuring these angles. ML: Medial Lateral Displacement (millimeters). AD: Angular Deformity (degrees). APD: Anterior Posterior Displacement (millimeters). GPA: Glenopolar Angle (degrees).

STUDY NUMBER	GPA (DEGREES)	ML DISPLACEMENT (MM)	AD (DEGREES)	AP DISPLACEMENT (MM)
01 SCAP FX	42	10	27	36
02 SCAP FX	17	14	42	7
03 SCAP FX	41	15	29	20
04 SCAP FX	46	15	34	14
05 SCAP FX	26	4	18	8

Scapula Fracture, Active vs. Passive (N=5)
